# Significant Broadband Photocurrent Enhancement by Au-CZTS Core-Shell Nanostructured Photocathodes

**DOI:** 10.1038/srep23364

**Published:** 2016-03-21

**Authors:** Xuemei Zhang, Xu Wu, Anthony Centeno, Mary P. Ryan, Neil M. Alford, D. Jason Riley, Fang Xie

**Affiliations:** 1 Department of Materials, Imperial College London, London, United Kingdom, SW7 2AZ; 2 Malaysia-Japan International Institute of Technology, University of Technology Malaysia International Campus, 54100, Kuala Lumpur, Malaysia

## Abstract

Copper zinc tin sulfide (CZTS) is a promising material for harvesting solar energy due to its abundance and non-toxicity. However, its poor performance hinders their wide application. In this paper gold (Au) nanoparticles are successfully incorporated into CZTS to form Au@CZTS core-shell nanostructures. The photocathode of Au@CZTS nanostructures exhibits enhanced optical absorption characteristics and improved incident photon-to-current efficiency (IPCE) performance. It is demonstrated that using this photocathode there is a significant increase of the power conversion efficiency (PCE) of a photoelectrochemical solar cell of 100% compared to using a CZTS without Au core. More importantly, the PCE of Au@CZTS photocathode improved by 15.8% compared to standard platinum (Pt) counter electrode. The increased efficiency is attributed to plasmon resonance energy transfer (PRET) between the Au nanoparticle core and the CZTS shell at wavelengths shorter than the localized surface plasmon resonance (LSPR) peak of the Au and the semiconductor bandgap.

Solar energy is the most abundant and sustainable natural energy source that could facilitate global sustainability. Photovoltaic cells in particular are a promising solar energy conversion technology that can directly convert solar light energy into electricity. Solar cells based on copper zinc tin sulfide (CZTS), a quaternary semiconducting compound, have been receiving increased interest as CZTS is composed of only abundant and non-toxic elements. Indeed it is estimated that only 1% of the global reserves of Cu, Sn, Zn and S could produce enough energy to power the world^1^.

CZTS is a promising light absorbing material^2^,^3^,^4^,^5^ with a high absorption coefficient (α > 10^4^ cm^−1^) and a direct bandgap of 1.4–1.6 eV^6^,^7^. Due to the p-type nature of CZTS, one area of interest is the incorporation of CZTS thin films as photocathodes in regenerative photoelectrochemical solar cells, such as dye-sensitized solar cells (DSSCs). In traditional DSSCs, platinum (Pt)-loaded conductive glass has been widely used as the standard counter electrodes^8^. However, high cost and low abundance greatly limit the large-scale use of Pt in DSSCs^9^. Photocathodes made from low-cost semiconductors as an alternative to Pt counter electrodes have attracted considerable attention, not only because the substitution of Pt may reduce the manufacturing cost of the devices, but also because the double-photoelectrode cells have the potential to harvest more light and give rise to higher current densities. To date, CZTS thin films have been successfully employed as photocathodes in photoelectrochemical solar cells^10^,^11^,^12^,^13^ and have shown increased cell efficiency relative to devices fabricated using Pt counter electrodes.

Plasmonic nanostructures such as copper, silver and gold have demonstrated enhanced performance for fluorescence based detection^14^,^15^,^16^, as well as for a photoanode in a photoelectrochemical cell^17^,^18^,^19^,^20^,^21^. Three possible enhancement mechanisms have been discussed in the literature^17^,^22^,^23^,^24^. These are plasmon resonance energy transfer^24^ (PRET), scattering effects and hot-carrier injection where the charge carrier gains sufficient kinetic energy to transfer from the metal nanoparticle to the conduction band of the semiconductor. In PRET the plasmonic metal absorbs photons and transfers the absorbed energy to the semiconductor *via* dipole-dipole coupling.

In this paper we consider whether the incorporation of metal nanoparticles into a CZTS photocathode can lead to an enhanced efficiency of the double-photoelectrode electrochemical solar cell. We demonstrate that coupling of the Au@CZTS photocathode with a dye-sensitized TiO_2_ photoanode and iodide/triiodide electrolyte leads to a cell that is 100% more efficient than a device fabricated in the same manner but with a photocathode made of CZTS only. Furthermore, the cell with Au@CZTS photocathode performs *ca.* 15.8% more efficient than using platinum as a counter electrode. To interrogate the mechanism for the enhanced cell performance electrochemical impedance spectroscopy experiments are undertaken. The optoelectronic effects of plasmonic metal nanoparticles in CZTS are discussed.

## Results and Discussion

XRD patterns of samples synthesized by reacting Cu-Zn-Sn metal precursor and thiol in/without the presence of Au nanoparticles are shown in Fig. S1 (Supplementary). The diffraction pattern of the sample prepared without Au can be identified as wurtzite CZTS. The pattern of the sample synthesized in the presence of Au consists of diffraction peaks from both Au and wurtzite CZTS. The diffraction peaks in both patterns are relatively broad, indicating the formation of nanosized particles.

**Figure 1:**
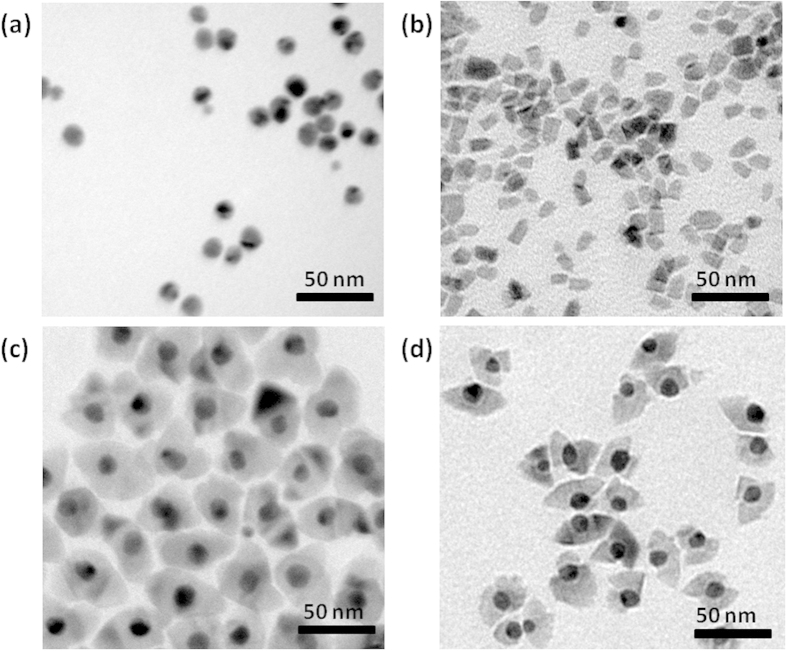
TEM image as-synthesized particles: (**a**) Au nanoparticles; (**b**) wurtzite CZTS nanoparticles; (**c**) TEM image of particles synthesized by reacting the original amount of metal precursors and thiol in the presence of Au nanoparticles; (**d**) TEM image of particles synthesized by reacting half amount of the original amount of metal precursors and thiol in the presence of Au nanoparticles.

To check if the particles prepared in the presence of Au have a core-shell structure or are just a mixture of individual Au and CZTS particles the morphology of as-prepared particles was investigated by TEM microscopy. TEM images of bare Au nanoparticles and pristine wurtzite CZTS nanoparticles are shown in Fig. 1a,b as references, respectively. TEM image of the sample synthesized by reacting metal precursors and thiol in the presence of Au nanoparticles (Fig. 1c) reveal that the resulting particles are not a mixture of individual Au particles and wurtzite CZTS particles but have a core-shell structure. As shown in the image, the gold core, of size 10–15 nm, is surrounded by a shell of CZTS which has a rice-like shape. With the aim of adjusting the thickness of the shell the amount of metal precursors was changed. Nanoparticles were synthesized when the metal precursors were reduced to half of the original concentration and the TEM image of the resultant material is shown in Fig. 1d. With the reduced precursor concentration most gold cores have incomplete shells, which is in contrast with the complete encapsulation of gold cores (Fig. 1c) when the samples were prepared at higher precursor concentration. This indicates that a suitable concentration of metal precursor is necessary for the formation of complete core-shell nanostructures.

To investigate the formation process of Au@CZTS core-shell nanostructure, aliquots were taken out from the reaction flask at different times after the injection of the sulfur source (thiol) into the mixture of Cu-Zn-Sn metal precursor and Au nanoparticles. The temperature was raised from 120 °C to 270 °C after the injection. TEM images of the particles extracted from the system at 2, 8, 18 and 30 minutes (end of the reaction) are shown in Fig. S2 (Supplementary). After 2 minutes (Fig. S2a), bare Au nanoparticles along with amorphous chunks of precursor are observed because the temperature of the system, 150 °C, was not thermodynamically high enough for the nucleation of CZTS. After 8 minutes (Fig. S2b), the temperature had reached 250 °C and core-shell nanostructure had formed. Prolonging reaction to 18 minutes and 30 minutes increases the average thickness of CZTS shell (Fig. S2c, d).

**Figure 2:**
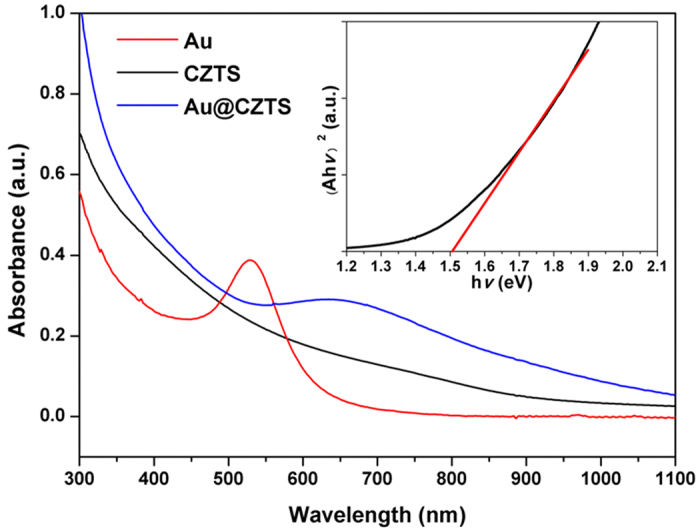
UV-Vis absorption spectra of as-synthesized nanoparticles suspended in toluene. Inset shows the plotting of (Ahν)^2^ *vs.* hν based on the UV-Vis absorption spectrum of CZTS which gives an estimation of its bandgap.

The optical property of the Au@CZTS core-shell nanostructure was investigated using UV-Vis spectroscopy (Fig. 2). Spectra of bare Au nanoparticles and pure wurtzite CZTS nanoparticles are both displayed for comparison. The spectrum of Au nanoparticles has a plasmonic peak centred at 530 nm; wurtzite CZTS shows a broad absorption extending to the edge of near infrared. The spectrum of Au@CZTS core-shell nanoparticles shows two features, a broad absorption and an Au plasmonic peak. The Au plasmonic peak in Au@CZTS nanoparticles shifts to *ca.* 680 nm, relative to 530 nm in the bare Au nanoparticles. This is consistent with the change in refractive index of the medium surrounding the gold, from toluene (~1.5) to CZTS (~2.5).

The bandgap of the wurtzite CZTS nanocrystals can be estimated by Tauc plot based on its UV-Vis absorption spectrum^25^. The estimated bandgap is *ca.* 1.51 eV by extrapolating the linear region of a plot of (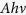)^2^ vs 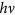 as shown in inset in Fig. 2. For Au@CZTS nanoparticles, the Au plasmon resonance precludes bandgap estimation from Tauc plot, however, similarity in the absorption edge indicates Au@CZTS has a similar bandgap as the pristine wurtzite CZTS material.

Au@CZTS thin films and CZTS thin films were made by spray-coating nanoparticle-toluene dispersion on clean substrates. The morphology of the thin films was observed by SEM (Fig. S3). Large cracks as wide as 300 nm can be observed in the films fabricated from pristine CZTS nanocrystals (Fig. S3a). The films made from Au@CZTS nanoparticles exhibit a more continuous surface, although narrower cracks are still visible in the film (Fig. S3b). This corresponds with the observation made by Aydil that thin films made from nanoparticles with larger size tend to have fewer cracks^26^. At higher magnification (Fig. S3c), it can be seen that the surface of the film is covered with Au@CZTS particles with a size of 30–50 nm, which agrees with the observed particle dimension observed in the TEM images (Fig. 1c). The cross-sectional image on the crack-free area of the film shows that the nanoparticles are densely packed (Fig. S3d).

**Figure 3:**
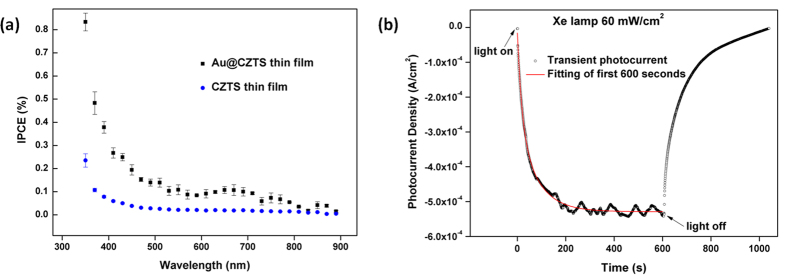
(**a**) IPCE curves of CZTS and Au@CZTS thin films under illumination from monochromatic light; (**b**) transient photocurrent of Au@CZTS thin film.

Au@CZTS thin films and CZTS thin films fabricated on FTO glass substrates were used as photoelectrodes in the three-electrode cell. Their IPCE curves are shown in Fig. 3a. Compared with the CZTS thin film, the Au@CZTS photocathode exhibits an enhanced IPCE across the entire wavelength range. It can be seen that there is a distinct peak between 600–750 nm which corresponds to the plasmon resonance peak of the gold core embedded in the CZTS matrix, as shown in the UV-Vis absorption spectrum (Fig. 2).

Transient photocurrent of an Au@CZTS photocathode was recorded in the same electrolyte under modulated 60 mW cm^−2^ radiation from a xenon lamp (Fig. 3b). The transient photocurrent has a negative sign which reflects the p-type nature of CZTS. At the onset of illumination the photocurrent increases slowly. The response (solid curve in Fig. 3b) fits a function which is the sum of two exponential components, with characteristic time of 18 s and 77 s. Chronoamperometric responses with two temporal components have been reported in studies of plasmonic photoanodes^27^. A maximum current of 540 μA cm^−2^ under illumination from a Xe lamp of 60 mW cm^−2^ indicates a higher photon-to-current conversion efficiency under more intense radiation than that observed in the previous monochromatic IPCE measurement, suggesting that charge carrier recombination *via* trap states occurs at low light intensity.

**Figure 4:**
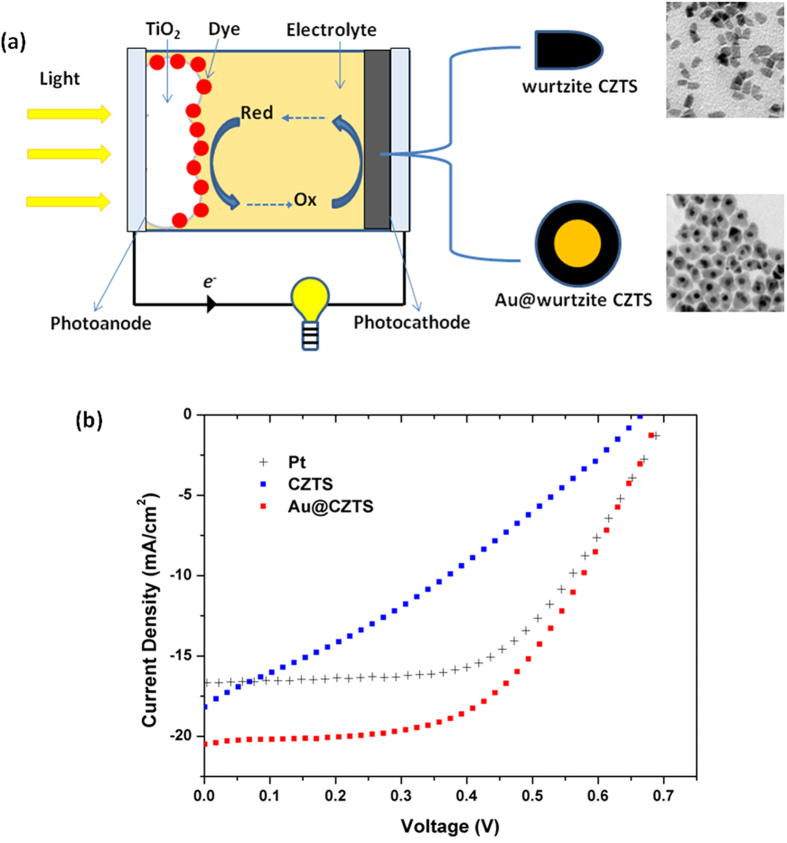
(**a**) Illustration of a photoelectrochemcal solar cell with a photocathode made from wurtzite CZTS/Au@wurtzite CZTS nanoparticles; (**b**) J–V curves of solar cells fabricated with different counter electrodes: Pt, CZTS and Au@CZTS.

Au@CZTS thin films and pristine wurtzite CZTS thin films (on FTO glass substrates) were employed as counter electrodes in photoelectrochemical solar cells as illustrated in Fig. 4a. A commercial platinum (Pt) counter electrode was also used as a comparison. Current-voltage (J–V) curves of three solar cells are shown in Fig. 4b and the key parameters summarized in Table 1.

As shown, in Table 1, solar cells fabricated with wurtzite CZTS thin films as the photocathodes both have higher short circuit photocurrent than a cell containing a Pt counter electrode. This is because CZTS acts as an absorbing layer and provides extra charge carriers, contributing to the photocurrent. However, the wurtzite CZTS photocathodes give rise to a slightly lower open circuit voltage and poorer fill factor, resulting in a lower cell efficiency compared with the cell consisting of a Pt counter electrode. On the other hand, solar cells fabricated with Au@CZTS films as the photocathodes, have similar open circuit voltage and fill factor to cells prepared using a Pt counter electrode. Solar cells with the Au@CZTS photocathodes possess higher short circuit current than the classic cell with a Pt counter electrode. The higher short circuit current means that the cell with an Au@CZTS counter electrode had an efficiency 15.8% higher than an identical cell with a Pt counter electrode, and twice that of pure wurtzite CZTS.

It should also be noted that kesterite phase CZTS counter electrodes have been reported with higher short circuit photocurrents than Pt^12^. In this work we attempted to synthesize Au@kesterite CZTS thin films but to date the quality of the particles have been poor. At present reasons are not clear, but the growth of CZTS on the surface of the Au may be considered as epitaxial and we think that there is a mismatch between the lattice parameter of the Au and kesterite CZTS. This is an area of continuing investigation which we shall endeavour to present results on in the future.

**Figure 5:**
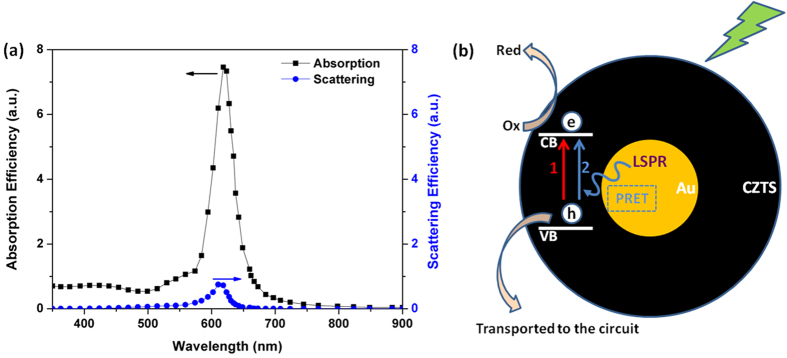
(**a**) Calculated absorption and scattering efficiency for a 15 nm diameter Au sphere embedded in wurtzite CZTS; (**b**) schematic of the charge carrier generation in Au@CZTS nanoparticles under illumination in two routes: Route 1 in red shows charge generation directly by incident light and Route 2 in blue shows charge generation by energy transfer from LSPR of Au core to CZTS *via* PRET.

To analyze the IPCE and associated UV-Vis curves further electromagnetic modelling was carried out using the Finite Difference code MEEP (MIT Electromagnetic Equation Propagation)^28^. The analysis consisted of calculating the absorption and scattering efficiency^29^ between 350–1000 nm, for a 15 nm diameter Au sphere surrounded by CZTS, using both the Finite Difference Time Domain (FDTD) and Frequency Domain (FDFD) solvers. The scattering efficiency of an object much smaller than the wavelength of the incident wave, is defined as the power scattered by the object divided by the power density of the incident wave on the cross-section. The absorption efficiency is found by considering the power dissipated, rather than scattered. (The calculation method and details of the electromagnetic modelling are more fully described in the supplementary information). The calculations showed the scattering efficiency to be very low across the spectrum compared to the absorption efficiency. The results are shown in Fig. 5a. It can be seen that there is a large absorption peak at 620 nm. It is also observable that there is higher absorption efficiency for wavelengths below 700 nm. The prepared CZTS has a bandgap of 1.51 eV, corresponding to a wavelength of 821 nm. The absorption peak at 620 nm of the Au nanoparticles means that there is a likelihood of non-radiative transfer of energy from the LSPR dipole of Au nanoparticles to the transition dipole of CZTS semiconductor to induce charge separation by plasmon resonant energy transfer (PRET). The strength of the PRET depends on the spectral overlap of the semiconductor’s band edge (absorption band) with the Au nanoparticle absorption and the distances between the two dipoles. It is noted that there is a large increase in IPCE below 500 nm for the Au@CZTS thin film (Fig. 3a), compared to the CZTS film. Previous literature has shown that there is coherent PRET at wavelengths below that of the donor dipole, the LSPR of the Au@CZTS^24^. This arises as there is still strong absorption of photons by the Au at these wavelengths and there remains an overlap with the absorption spectra of the CZTS. The enhancement at short wavelengths is consistent with the work recently reported by Li *et al.*^24^ We also note that non-plasmonic enhancement of photocurrent, due to Au nanoparticles mixed with Copper (Cu) based nanocrystals, has recently been reported^30^. This is due to the effect of an enhanced magnetic field on the Cu-based delofassite nanocrystals. Therefore, this is not the enhancement mechanism in CZTS.

CZTS, as a new solar cell material, has recently attracted a lot of research interest due to its ability to generate stable and high quality photocurrent from solar illumination. The efficient separation of excited electrons from holes over CZTS could reduce the radiative recombination of excitons and generate photocurrent for external circuitry^31^. The introduction of Au core into the CZTS can clearly generate more photoelectrons for both photovoltaic and catalytic hydrogen production applications^31^. A proposed pathway for photocurrent generation in the photoelectrochemical cells is described as follows (shown in Fig. 5b). When the Au@CZTS particles were employed as a photocathode material in the photoelectrochemical solar cells, light which passed through the photoanode and electrolyte could excite electrons from the valence band of CZTS into the conduction band (Route 1 in Fig. 5b), which contributed higher photocurrent to the circuit compared with Pt counter electrode. Furthermore, the Au nanoparticles can transfer energy to CZTS via PRET (Route 2 in Fig. 5b), contributing extra charge carriers and hence a higher photocurrent relative to pure CZTS photocathode. The produced photoelectrons on the sulphide surface are captured by oxidized species in the electrolyte, while the holes are transported to the external circuit to complete the photoelectrochemical cycle. Electrochemical impedance spectroscopy (EIS) studies suggest that the enhancement arises due to increased absorption at the photocathode rather than increased scattering (Details of the EIS is given in the Supplementary). This is consistent with the electromagnetic modelling data where very low scattering efficiency was predicted across the spectrum.

## Conclusion

We have demonstrated significant and broadband IPCE enhancement of the photoelectrode containing Au@CZTS core-shell nanostructures, compared to CZTS without Au core. When the Au@CZTS photocathode was employed as the counter electrode in a photoelectrochemical solar cell, the power conversion efficiency of the cell was doubled, compared to an identical cell with CZTS as a counter electrode. Moreover, the power conversion efficiency of the Au@CZTS photocathode was 15.8% higher than a standard Pt counter electrode. In summary, we circumvent the limitations of CZTS by introducing the novel Au@CZTS core-shell nanostructrure, potentially paving the way for large-scale use of CZTS in solar cells.

## Methods

### Synthesis of Au@CZTS Nanoparticles and CZTS Nanoparticles

The Au@CZTS core-shell nanoparticles were synthesized adapting a reported method from literature^31^. To start with, Cu-Zn-Sn metal precursor solution was prepared in a three-neck bottle attached to a Schlenk line. In a typical synthesis, 1.8 mmol copper acetylacetonate (Cu(acac)_2_), 1.2 mmol zinc acetate (Zn(OAc)_2_) and 1.0 mmol tin chloride (SnCl_2_) were mixed with 5 cm^3^ oleylamine. The mixture was then heated to 120 °C under argon and stirred for 30 minutes to dissolve the metal salts. Au nanoparticles as the core of the heterostructures were prepared separately on a hot plate. 0.05 mmol HAuCl_4_·3H_2_O was dissolved in 5 cm^3^ oleylamine in a glass vial, heated to 120 °C and stirred for 10 minutes for Au nanoparticle growth. The Au nanoparticles were quickly injected into the Cu-Zn-Sn metal precursor in the flask. The mixture was stirred for 5 minutes to allow homogenous dispersion of Au nanoparticles in the precursor solution. 0.05 cm^3^ 1-dodecanethiol (1-DDT) and 0.35 cm^3^ *tert*-dodecanethiol (*t*-DDT) was then injected into the mixture simultaneously and temperature was increased to 270 °C in *ca.* 10 minutes. After 30 minutes, the heating mantle was removed and the mixture was cooled down to room temperature. The nanoparticles were separated from the liquid phase by centrifuging followed by washing with toluene and isopropanol three times. The nanoparticles were dispersed in toluene by sonication for future use. In order to obtain different thicknesses of CZTS on the Au core the amounts of Cu, Zn, and Sn salts were varied at constant mole ratios for the preparation of Cu-Zn-Sn precursor solution.

To investigate the influence of Au incorporation on the properties of nanoparticles, pristine CZTS nanocrystals were synthesized as a blank control using an identical procedure to the above but omitting the addition of Au nanoparticles and using a reaction time of 1 hr.

### Thin film Fabrication

Au@CZTS/CZTS nanoparticles were dispersed in toluene under sonication to form a suspension which had a concentration of 10–20 mg cm^−3^. Nanoparticle suspension was then sprayed onto FTO glass substrates, pre-cleaned using UV-Ozone, by an airbrush with an argon flow at a spray rate of 0.1–0.3 cm^3^ min^−1^. During the spray-coating, the nozzle to substrate distance was maintained at 10–15 cm and the substrate temperature was *ca.* 100 °C. The films were dried in a vacuum oven at 160 °C for 2 hr. The pristine and core-shell nanoparticle films which were only dried in the oven without heat treatment were labelled as ‘CZTS’ and ‘Au@CZTS’, respectively.

## Characterization

The morphology of nanoparticles was observed using a JEOL 2000FX transmission electron microscope (TEM) operated with an accelerating voltage of 300 kV. TEM samples were prepared by drop-casting dilute nanoparticle-toluene dispersion onto carbon-coated copper TEM grids (300 mesh, Agar Scientific UK). The morphology of thin films were observed using a LEO Gemini 1525 field emission gun scanning electron microscope (FEGSEM) with an accelerating voltage of 5 kV. The films were made by spray-coating nanoparticles on glass substrates and then the glass substrates were mounted on aluminium stubs (Agar Scientific UK) by double-sided carbon conductive adhesive tape (Agar Scientific UK). The morphology of nanoparticles and thin films were observed using a JEOL 2000FX transmission electron microscope (TEM) and a LEO Gemini 1525 field emission gun scanning electron microscope (FEGSEM). X-ray diffraction (XRD) studies of the particles were performed on a Bruker D2 diffractometer with monochromated Cu Kα source. Ultraviolet-visible (UV-Vis) spectra of the sample were measured using a Perkin Elmer, LambdaBIo10 spectrometer. Monochromatic incident photon to current efficiency (IPCE) spectra of the as-prepared thin films were recorded in a three-electrode photoelectrochemical cell. The electrolyte was 0.1 mol dm^−3^ EuCl_3_ with 0.1 mol dm^−3^ KCl. A saturated Ag/AgCl electrode and a platinum mesh electrode were used as the reference electrode and counter electrode respectively. A μ-Autolab potentiostat coupled to a PTi OC-4000 optical chopper and a SR830 lock-in amplifier at a reference frequency of about 0.05 Hz was used to record the photocurrent density at a monochromatic wavelength λ and a potential of −0.5 V *vs.* saturated Ag/AgCl reference electrode. The incident light power intensity was monitored by a Thorlabs PM100A light power meter.

### Solar Cell Fabrication and Characterization

To test the performance of Au@CZTS and CZTS nanoparticles as photocathode material, Au@CZTS and CZTS thin films were incorporated in to devices fabricated using a commercial DSSC kit (Solaronix). Titania thin films (Solaronix) were immersed in methanol containing 0.3 mmol dm^−3^ ruthenizer 535-bisTBA (N719, Solaronix) for 8 hr. The dye-stained titania thin films were used as photoanodes and assembled with various counter electrodes, *i.e.*, CZTS nanoparticles thin films on FTO glass, Au@CZTS thin films on FTO glass and platinized FTO, using a 100 μm thick Surlyn^®^ thermoplastic sealing gasket film (Solaronix, Meltonix 1170–100). An iodide/tri-iodide electrolyte (AN-50, Solaronix) was then injected into the gap between the two electrodes. Finally a black mask was attached to allow a 0.25 cm^2^ active area. The steady state current density-voltage (J–V) curves were recorded using a Keithley 2400-LV source meter under simulated solar light illumination with an intensity of 100 mW cm^−2^ (1 sun), generated by a certified AAA solar simulator (Newport, LCS-100TM, 94011A) with an AM 1.5 G filter. Electrochemical impedance spectroscopy studies were performed on the as-prepared cells under AM 1.5 G illumination and a DC voltage bias of −600 mV *vs.* the open circuit voltage, an AC modulation of 0.01 V was applied.

## Additional Information

**How to cite this article**: Zhang, X. *et al.* Significant Broadband Photocurrent Enhancement by Au-CZTS Core-Shell Nanostructured Photocathodes. *Sci. Rep.* **6**, 23364; doi: 10.1038/srep23364 (2016).

## Supplementary Material

Supplementary Information

## Figures and Tables

**Table 1:** Key parameters of DSSCs extracted from Fig. 4.

Counter electrode	V_OC_(V)	J_SC_ (mA/cm^2^)	FF	η (%)
Pt	0.70	16.59	0.57	6.64
CZTS	0.67	18.18	0.31	3.72
Au@CZTS	0.70	20.25	0.54	7.69

## References

[b1] Wadia C., Alivisatos A. P. & Kammen D. M.. Materials availability expands the opportunity for large-scale Photovoltaics Deployment. Environmental Science & Technology 43, 2072–2077 (2009). 19368216 10.1021/es8019534

[b2] Shin B. *et al.* Thin film solar cell with 8.4% power conversion efficiency using an earth‐abundant Cu_2_ZnSnS_4_ absorber. Progress in Photovoltaics: Research and Applications 21, 72–76 (2013).

[b3] Repins I. *et al.* Co-evaporated Cu_2_ZnSnSe_4_ films and devices. Solar Energy Materials and Solar Cells 101, 154–159 (2012).

[b4] Guo Q. *et al.* Fabrication of 7.2% efficient CZTSSe solar cells using CZTS nanocrystals. Journal of the American Chemical Society 132, 17384–17386 (2010). 21090644 10.1021/ja108427b

[b5] Wang W. *et al.* Device characteristics of CZTSSe thin-film solar cells with 12.6% efficiency. Advanced Energy Materials 4, 1301465 (2014).

[b6] Katagiri H. *et al.* Development of thin film solar cell based on Cu2ZnSnS4 thin films. Solar Energy Materials and Solar Cells 65, 141–148 (2001).

[b7] Katagiri H.. Cu_2_ZnSnS_4_ thin film solar cells. Thin Solid Films 480, 426–432 (2005).

[b8] Murakami T. N. & Grätzel M.. Counter electrodes for DSC: application of functional materials as catalysts. Inorganica Chimica Acta 361, 572–580 (2008).

[b9] Hou Y. *et al.* Rational screening low-cost counter electrodes for dye-sensitized solar cells. Nat. Commun. 4, 1583 (2013). 23481391 10.1038/ncomms2547

[b10] Xin X., He M., Han W., Jung J. & Lin Z.. Low-cost copper zinc tin sulfide counter electrodes for high-efficiency dye-sensitized solar cells. Angewandte Chemie International Edition 50, 11739–11742 (2011). 10.1002/anie.201104786 21898741

[b11] Zeng X. *et al.* Low-cost porous Cu_2_ZnSnSe_4_ film remarkably superior to noble Pt as counter electrode in quantum dot-sensitized solar cell system. Journal of Power Sources 226, 359–362 (2013).

[b12] Xu J., Yang X., Yang Q. D., Wong T. -L. & Lee C. S.. Cu_2_ZnSnS_4_ hierarchical microspheres as an effective counter electrode material for quantum dot sensitized solar cells. Journal of Physical Chemistry C 116, 19718–19723 (2012).

[b13] Chen H. *et al.* Effect of crystallization of Cu_2_ZnSnSxSe_4_–x counter electrode on the performance for efficient dye-sensitized solar cells. ACS Applied Materials & Interfaces 6, 20664–20669 (2014). 25382857 10.1021/am503963b

[b14] Xie F., Centeno A., Ryan M. R., Riley D. J. & Alford N. M.. Au nanostructures by colloidal lithography: from quenching to extensive fluorescence enhancement. Journal of Materials Chemistry B 1, 536–543 (2013). 10.1039/c2tb00278g 32260825

[b15] Xie F. *et al.* Nanoscale control of Ag nanostructures for plasmonic fluorescence enhancement of near-infrared dyes. Nano Research 6, 496–510 (2013).

[b16] Goldysm E. M. & Xie F.. Metallic nanomaterials for sensitivity enhancement of fluorescence detection. Sensors 8, 886–896 (2008) 10.3390/s8020886 PMC3927526 27879741

[b17] Qi J., Dang X., Hammond P. T. & Belcher A. M.. Highly efficient plasmon-enhanced dye-sensitized solar cells through metal@oxide core-shell nanostructure. ACS Nano 5, 7108–7116 (2011). 21815674 10.1021/nn201808g

[b18] Kumar P. N., Deepa M. & Ghosal P.. Low-cost copper nanostructures impart high efficiencies to quantum dot solar cells. ACS Applied Materials & Interfaces 7, 13303–13313 (2015). 26000891 10.1021/acsami.5b01175

[b19] Zarick H. F. *et al.* Enhanced efficiency in dye-sensitized solar cells with shape-controlled plasmonic nanostructures. ACS Photonics 1, 806–811 (2014).

[b20] Yun J., Hwang S. H. & Jang J.. Fabrication of Au@Ag core/shell nanoparticles decorated TiO_2_ hollow structure for efficient light-harvesting in dye-sensitized solar cells. ACS Applied Materials & Interfaces 7, 2055–2063 (2015). 25562329 10.1021/am508065n

[b21] Wu X. *et al.* Broadband plasmon photocurrent generation from Au nanoparticles/mesoporous TiO_2_ nanotube electrodes. Solar Energy Materials and Solar Cells 138, 80–85 (2015)

[b22] Warren S. C. & Thimsen E.. Plasmonic solar water splitting. Energy & Environmental Science 5, 5133–5146 (2012).

[b23] Xuming Z., Yu Lim C., Ru-Shi L. & Din Ping T.. Plasmonic photocatalysis. Reports on Progress in Physics 76, 046401 (2013). 23455654 10.1088/0034-4885/76/4/046401

[b24] Li J. *et al.* Plasmon-induced resonance energy transfer for solar energy conversion. Nature Photonics 9, 601–607 (2015).

[b25] Tauc J. & Menth A.. State in the gap. Journal of Non-Crystalline Solids 8–10, 569–585 (1972).

[b26] Chernomordik B. D. *et al.* Rapid facile synthesis of Cu_2_ZnSnS_4_ nanocrystals. Journal of Materials Chemistry A 2, 10389–10395 (2014).

[b27] Mubeen S. *et al.* An autonomous photosynthetic device in which all charge carriers derive from surface plasmons. Nat Nano 8, 247–251 (2013). 10.1038/nnano.2013.18 23435280

[b28] Oskooi A. F. *et al.* Meep: A flexible free-software package for electromagnetic simulations by the FDTD method. Computer Physics Communications 181, 687–702 (2012).

[b29] Centeno A, Ahmed B, Reehal H & Xie F. Diffuse scattering from hemispherical nanoparticles at the air-silicon interface. Nanotechnology 24, 415402 (2013). 24045859 10.1088/0957-4484/24/41/415402

[b30] Xu X. *et al.* Near Field Enhanced Photocurrent Generation in P-type Dye-Sensitized Solar Cells. Scientific Reports 4, 3961 (2014). 24492539 10.1038/srep03961 PMC3912483

[b31] Ha E. *et al.* Significant Enhancement in Photocatalytic Reduction of Water to Hydrogen by Au/Cu_2_ZnSnS_4_ Nanostructure. Advanced Materials 26, 3496–3500 (2014). 24644004 10.1002/adma.201400243

