# Supplementary material for: Significant Broadband Photocurrent Enhancement by Au-CZTS Core-Shell Nanostructured Photocathodes
Source: Sci Rep. 2016 Mar 21;6:23364. doi: 10.1038/srep23364 (PMC4800310; doi:10.1038/srep23364)
Supplement: Supplementary Information [file srep23364-s1.pdf]

Supplementary Information

Significant Broadband Enhancement of Photoelectrochemical Cells by Au-CZTS Core-Shell Nanostructured Photocathodes

Xuemei Zhang[1,a], Xu Wu[1,a], Anthony Centeno[2], Mary P. Ryan[1], Neil M. Alford[1], D. Jason Riley[1], Fang Xie[1,*]

1, Department of Materials, Imperial College London, London, United Kingdom, SW7 2AZ

a, These authors contributed equally to this work and are co-first authors

2, Malaysia-Japan International Institute of Technology, University of Technology Malaysia International Campus, 54100, Kuala Lumpur, Malaysia

*** Corresponding author: Dr. Fang Xie: f.xie@imperial.ac.uk**

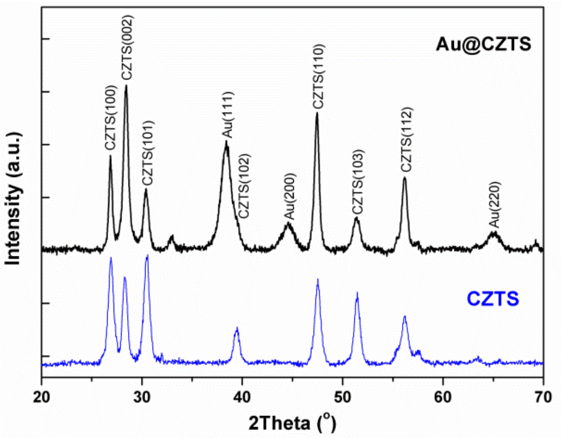

Fig. S1. XRD patterns of samples prepared by reacting Cu-Zn-Sn metal precursor and thiol in/without the presence of Au nanoparticles.

For CZTS, wurtzite is a relatively new crystal structure. Therefore, wurtzite CZTS cannot be found in the XRD database, but there has been a few works which show stimulated XRD pattern of wurtzite CZTS [1,2]. The diffraction peaks indexing of wurtzite CZTS in this work is referred to these works.

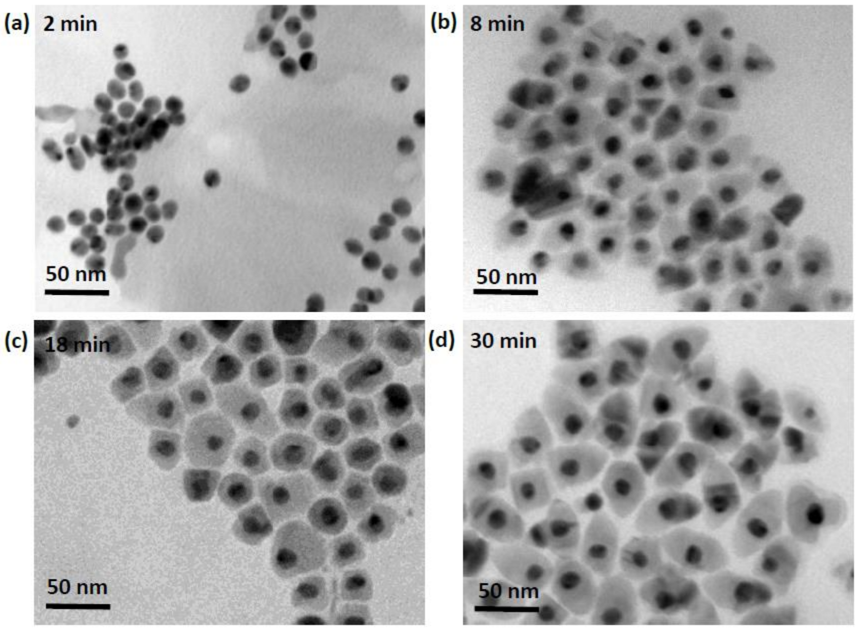

Fig. S2. TEM images of particles taken out from the reaction flask at different time when thiol was used the sulfur source: (a) 2 min; (b) 8 min; (c) 18 min; (d) 30 min (end of the reaction); (d) TEM images of the particles after reaction of 30 min when element sulfur powder was used as the sulfur source.

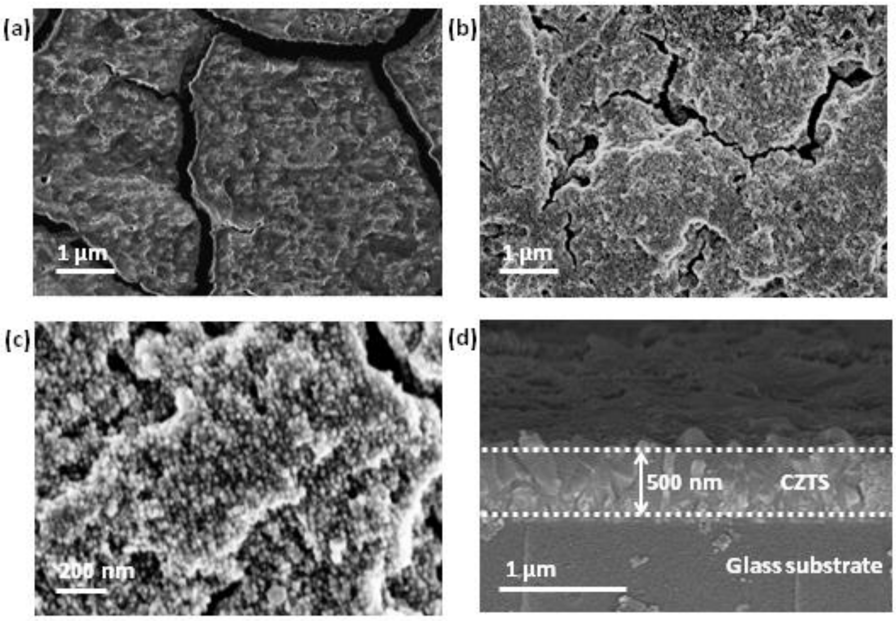

Fig. S3. SEM of images: (a) top view of a thin film made from wurtzite CZTS nanoparticles; (b) top view of a thin film made from Au@CZTS nanoparticles; (c) top view of an Au@CZTS film at a higher magnification; (d) cross-section of a thin film made from Au@CZTS nanoparticles on a glass substrate.

ElS Studies The electrochemical impedance spectra (EIS) were measured under AM 1.5 at -600 mV *vs.* open circuit for two devices, one with a pristine wurtzite CZTS photocathode and the other with an Au@CZTS photocathode. The two spectra are shown in Fig. S4. The EIS data was fitted using the equivalent circuit shown [3] and the best fit values obtained given in Table S1. It is observed that the cell with an Au@CZTS photocathode exhibited a significantly smaller R2 value. By varying the parameters in the equivalent circuit, between the values obtained for both cases, it was observed that R2 was the component that had the most effect on the Nyquist plot. This is illustrated in Figure S5 where the value of R2 is changed from 94.46 Ohms to 39.51 Ohms, whilst the other values are the same as the wurtzite CZTS counter electrode model. The smaller value of R2 points to a higher photocurrent at the Au@CZTS photocathode, than at the CZTS photocathode, and that this is the dominant effect of adding the Au. This result indicates that the enhancement is caused predominantly by localised effects in the photocathode, due to that addition of the Au nanoparticles.

Other components in the equivalent circuit are seen to be modified by the Au cores and have an effect on the high frequency and Warburg impedance. This may be caused by the scattering peak seen at the LSPR wavelength, Figure 5(a), but the fact that the main effect of the Au cores is seen by a change in R2 provides some indication that increased absorption by the Au is dominant, since light scattered from gold cores might be expected to have more influence on both electrodes. This is consistent with the results of the electromagnetic modelling, where the scattering efficiency was seen to be very small compared to the absorption efficiency.

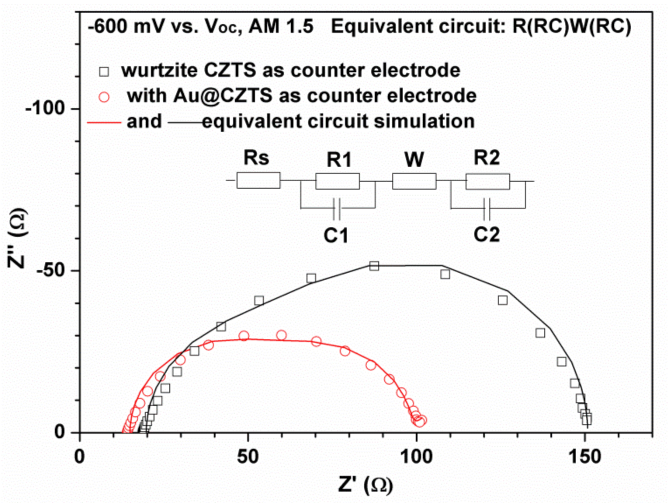

Fig. S4. Nyquist plots of solar cells with different counter electrodes (frequency range 10^6-10^0 Hz, geometric area was 0.25 cm^2), and insert figure is the diagram of Equivalent Circuit employed to simulate the impedance spectra.

Table S1. Fitting parameters of the Equivalent Circuit of Nyquist plots in Fig. S4.

Fitting parameters (Unit)	Counter electrode	
	CZTS	Au@CZTS
R_s (Ω)	19.63	14.86
R_1 (Ω)	34.75	43.20
C_1 (F)	9.41×10^{-6}	5.41×10^{-6}
R_2 (Ω)	94.46	39.51
C_2 (F)	2.31×10^{-5}	3.78×10^{-5}
W (S $s^{1/2}$)	4.10	10.28

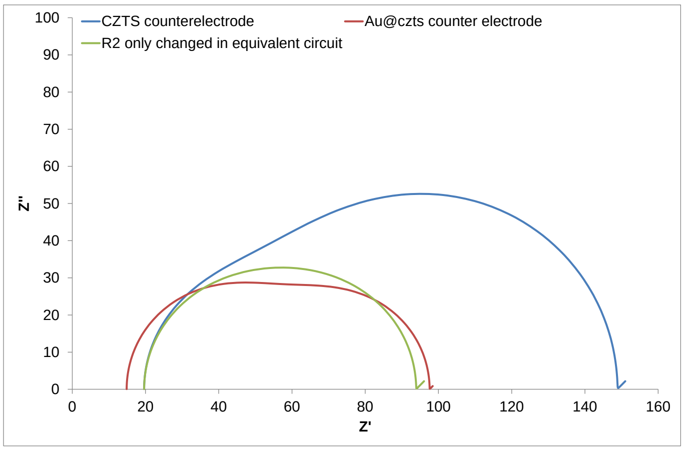

Fig. S5. Effect of changing R2, in the equivalent circuit, on the Nyquist plot.

Electromagnetic Modelling

Electromagnetic modelling was carried out using the Finite Difference code MEEP (MIT Electromagnetic Equation Propagation) [4]. The analysis consisted of calculating the absorption and scattering efficiency [5] between 350 nm and 1 µm, for a 15 nm diameter Au sphere surrounded by CZTS, using both the Finite Difference Time Domain (FDTD) and Frequency Domain (FDFD) solvers available in MEEP.

In the finite difference (FD) electromagnetic model the mesh discretization was found by convergence analysis and a mesh resolution, Δx, of 1 nm was required in both solvers. The time step, Δt, is given by $\Delta t = S\,\Delta x$, where S is the Courant factor. In our analysis we take S to be 0.5. The FD workspace was terminated in all directions with a perfectly matched layer to prevent non-physical reflections. In the FDTD calculations the excitation was a Gaussian pulse. In the FDFD calculations the excitation was a fixed frequency source at the wavelength of the calculation.

The dielectric function for Au is described using a Drude-Lorentz model, with one Drude term and five Lorentz terms in the summation [5]. The dielectric function for CZTS can be described using a Tauc-Lorentz oscillator model [6]. Whilst the Drude-Lorentz model is implemented and has been extensively used, the Tauc-Lorentz model, unfortunately, has yet to be realized in the MEEP FDTD code. For wavelengths longer than 600 nm the CZTS was modelled with real dielectric constant of 6 and a single FDTD calculation was carried out.

Below 600 nm a number of FDFD calculations were performed, since the absorption of CZTS becomes significant, and so the complex part of the permittivity cannot be ignored. Between 350 nm and 600 nm the complex permittivity of Au was calculated from the Drude-Lorentz model. For CZTS the real and imaginary parts of dielectric constant at 300 K were taken from the results presented by Li *et al.* [6]. The complex permittivities were then used in the FDFD solver to find the absorption and scattering efficiency. The imaginary part of the dielectric functions being simulated by setting an equivalent conductivity for both Au and CZTS at the wavelength of calculation. FDFD calculations were carried out for fifty wavelengths between 350 nm and 600 nm and combined with the FDTD calculations, for 15 nm diameter Au spheres embedded into CZTS, to produce Figure 5a in the paper.

The absorption efficiency, of an object much smaller than the wavelength, is defined as the power dissipated in the object under plane wave illumination, divided by the power density of the incident wave on the cross-sectional area, S_i. Similarly the scattering cross section is defined by considering the power scattered from the object. If we consider a surface totally enclosing the scattering object

then we can calculate the the normalized absorption (Q_{abs}) and scattering (Q_{sca}) efficiencies from accroding to the reported method[5]:

$$Q_{abs} = \frac{\int_s \mathrm{Re}\left(\frac{1}{2}\vec{E}\times\vec{H}^*\right)}{|S_i|}d\vec{a} \tag{1}$$

$$Q_{sca} = \frac{-\int_s \mathrm{Re}\left(\frac{1}{2}\vec{E}_S\times\vec{H}_S{}^*\right)}{|S_i|}d\vec{a} \tag{2}$$

The subscript s denotes the scattered, rather than the total field, at the surrounding surface. Using computational electromagnetics, such as FDTD or FDFD, to calculate the electric (*E*) and magnetic (*H*) fields at the enclosing surface equations (1) and (2) can be solved.

It should be noted that in the calculations we considered that CZTS fully surrounded the Au particle. The scenario considered was Au embedded in a CZTS material, that is as placed within a homogeneous thin film, rather than an isolated shell surrounded by air. We considered that this was much closer to the Au@CZTS counter electrode obtained from the experimental work and for which measurements were taken.

References

1. X. Lu, Z. Zhuang, Q. Peng and Y. Li, Chem. Commun., 2011, 47, 3141-3143.

2. M. Li, W. Zhou, J. Guo, Y. Zhou, Z. Hou, J. Jiao, Z. Zhou, Z. Du, and S. Wu, Journal of Physical Chemistry C, 2012, 116, 26507-26516.

3. T. N. Murakami and M. Grätzel, Inorganica Chimica Acta, 2008, 361, 572-580.

4. A.F. Oskooi, D.Roundy, M.Ibanescu, P.Bermel, J.D.Joannopoulos and S.G.Johnson, Computer Physics Communications, 2012, 181, 687-702.

5. A.Centeno, B.Ahmed, H.Reehal and F.Xie, Nanotechnology, 2013, 24, 41, 415402.

6. W. Li, K. Jiang, J. Zhang, X Chen, Z. Hu, S. Chen, L.Sun and J. Chu, Phys. Chem. Chem. Phys, 2012, 14, 9936-9941.
